# Genetic background influences murine prostate gene expression: implications for cancer phenotypes

**DOI:** 10.1186/gb-2007-8-6-r117

**Published:** 2007-06-18

**Authors:** Daniella Bianchi-Frias, Colin Pritchard, Brigham H Mecham, Ilsa M Coleman, Peter S Nelson

**Affiliations:** 1 Divisions of Human Biology and Clinical Research, Fred Hutchinson Cancer Research Center, Fairview Avenue, Seattle, WA 98109-1024, USA

## Abstract

**Background:**

Cancer of the prostate is influenced by both genetic predisposition and environmental factors. The identification of genes capable of modulating cancer development has the potential to unravel disease heterogeneity and aid diagnostic and prevention strategies. To this end, mouse models have been developed to isolate the influences of individual genetic lesions in the context of consistent genotypes and environmental exposures. However, the normal prostatic phenotypic variability dictated by a genetic background that is potentially capable of influencing the process of carcinogenesis has not been established.

**Results:**

In this study we used microarray analysis to quantify transcript levels in the prostates of five commonly studied inbred mouse strains. We applied a multiclass response *t*-test and determined that approximately 13% (932 genes) exhibited differential expression (range 1.3-190-fold) in any one strain relative to other strains (false discovery rate ≤10%). Expression differences were confirmed by quantitative RT-PCR, or immunohistochemistry for several genes previously shown to influence cancer progression, such as *Psca*, *Mmp7*, and *Clusterin*. Analyses of human prostate transcripts orthologous to variable murine prostate genes identified differences in gene expression in benign epithelium that correlated with the differentiation state of adjacent tumors. For example, the gene encoding apolipoprotein D, which is known to enhance resistance to cell stress, was expressed at significantly greater levels in benign epithelium associated with high-grade versus low-grade cancers.

**Conclusion:**

These studies support the concept that the cellular, tissue, and organismal context contribute to oncogenesis and suggest that a predisposition to a sequence of events leading to pathology may exist prior to cancer initiation.

## Abstract 2

Microarray analyses to quantitate transcript levels in the prostates of five inbred mouse strains identified differences in gene expression in benign epithelium that correlated with the differentiation state of adjacent tumors.

## Background

Family history and race represent two of the greatest contributors to the probability of developing cancer of the prostate. Recent estimates suggest that 42% of prostate cancer risk may be attributed to heritable factors that include the influence of rare alleles capable of exerting substantial effects, common alleles with weak effects, and gene interactions that act to amplify or buffer phenotypes [1]. Racial background accounts for disparities of more than 40-fold in the incidence of prostate cancer between Western and Asian men, and also associates with cancer progression and lethality [2]. Importantly, risks attributed to racial categories may reflect not only genetic variables, but also a myriad of shared environmental exposures that include diet, infectious disease, and medication use.

Cancer susceptibility represents a continuum of interactions between the host and environment. At the extremes, each can exert dominant effects on the neoplastic process. For example, inherited differences in specific gene products, such as p53, Rb, and APC, lead to the near-universal development of cancers, regardless of differences in the host environment [3]. Similarly, exposures to ionizing radiation or chemical mutagens can produce high rates of neoplasia regardless of the host genetic background. However, most human malignancies cannot be attributed to specific genes or extrinsic agents that exert dominant effects, but rather arise in the setting of complex multi-factorial gene-environment relationships. In this context, studies of twins have found that genetic background is associated with a large proportion of supposedly nonhereditary cancers, a finding supported by the familial clustering of specific malignancies [1].

The identification of low-penetrance genetic modifiers that influence cancer phenotypes has been challenging in humans due to substantial genetic heterogeneity and the inability to identify, quantify and control for a wide-range of environmental variables. Furthermore, tumors arising in specific organ sites may exhibit multiple different histologies that include differentiation state and the propensity to progress at variable rates [4,5]. To overcome these hurdles, inbred strains of model organisms such as the mouse have been used to control environmental influences, homogenize tumor histologies, and reduce the complexity of genetic backgrounds [6]. Manipulating these variables has facilitated studies that link genomic loci with the propensity to develop neoplasia and the identification of genes that modulate tumor behavior. Despite highly similar genomes, striking differences in tumorigenesis and metastasis have been observed in different rodent strains induced to develop cancers of the lung, breast, intestine, skin, and prostate [7-11]. Breeding strategies designed to isolate the genes responsible for cancer susceptibility have successfully identified modifying loci [12]. The characterization of specific genes modulating cancer phenotypes indicates that carcinogenesis is influenced by tumor-intrinsic features as well as variables in the host macro- and microenvironments [13]. Intrinsic cellular properties include proliferation rates, genome stability, differentiation potential and the ability to senesce or undergo apoptosis. Tumor-'extrinsic' factors that influence the process of carcinogenesis include hormone concentrations, immune response, drug metabolism, and features of the local stroma involving matrix and neovascularization. Importantly, many cancer-modifying loci exhibit multiple genetic interactions that suggest the existence of molecular networks that underlie cancer predisposition [6,7].

Studies of prostate carcinogenesis in rodent models developed using chemical mutagens or gene-targeting strategies have clearly demonstrated modifications of cancer incidence and progression rates dependent on the host genotype. The substantial tumor-promoting or tumor-suppressing effects exerted by innate host factors suggests that features of benign tissues could allow the behavior of tumor growth to be predicted. To support this hypothesis, influential biochemical or tissue variations must occur and must exhibit measurable characteristics. While variations in immune effectors and hormone levels represent likely influences on prostate carcinogenesis in these model systems, differences intrinsic to the prostate gland could also account for tumor incidence rates between strains. One measurement of phenotypic potential involves the identification and quantification of cellular gene transcription.

To date, global analyses of gene expression in the normal prostate gland of mouse strains have not been reported. In this study, we used microarray analysis to profile prostate gene expression across five inbred mouse strains commonly used for modeling prostate development and carcinogenesis. We found substantial strain-dependent differences in prostate transcript expression patterns, including several genes implicated in prostate cancer development and progression. Analyses of these strain-variable genes in the human prostate enabled the determination of associations between transcript expression levels and phenotypes of prostate cancer, such as tumor grade. The results indicate that variables in prostate gene expression present prior to cancer initiation could modify tumorigenesis.

## Results and discussion

### Determination of strain-specific differences in mouse prostate gene expression

Several studies have demonstrated the influence of genetic background on the development and progression of prostate cancer in rodents. Using a genetically engineered mouse model driving SV40T antigen expression in the prostate gland, designated TRAMP, Gingrich *et al*. [14] determined that prostate tumors arising in a mixed C57BL/6 × FVB background display reduced latency, increased primary tumor growth and enhanced metastatic progression when compared to tumor development in a pure C57BL/6 background. A recent study of *Pten *deficient mice reported a critical role for genetic background that influenced the onset, tumor spectrum, and progression rates for cancers that included prostate carcinoma [15]. Strain-specific effects have also been observed in mice with inactivation of the prostate-specific *Nkx3.1 *homeobox gene: the occurrence of intraepithelial neoplasia was more frequent in C57BL/6 and FVB/N strains than in the 129/SvImJ background (Cory Abate-Shen, personal communication). Genetic background has also been reported to influence transgenic models of rat prostate carcinogenesis, with cancer incidence rates ranging from 0% to 83%, depending on strain background [11].

**Figure 1:**
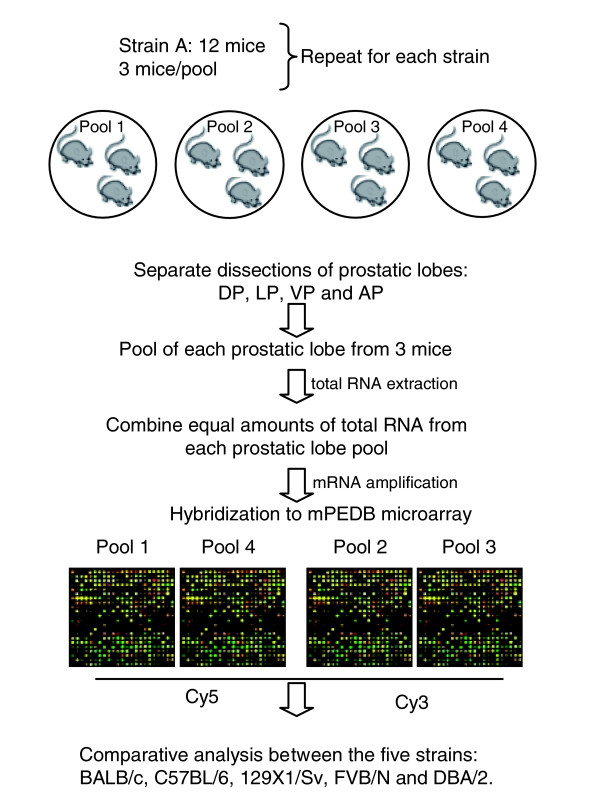
Experimental design. Prostates from 12 mice from each of 5 strains of *Mus musculus *(C57BL/6, 129X1/Sv, BALB/c, FVB/N and DBA/2) were resected and individual lobes were dissected: DP, dorsal prostate; LP, lateral prostate; VP, ventral prostate; AP, anterior prostate. Each experimental sample represents a pool of equal amounts of RNA for each prostatic lobe from three animals. Four independent experimental samples were created per strain: 12 mice divided into 4 pools of 3 mice each for a total of 4 microarray experiments per strain. Amplified RNA from each experimental sample was hybridized against a reference pool onto custom mouse prostate cDNA microarrays using alternate dye-labeling to account for dye-specific effects.

To ascertain the extent of gene expression variability in the normal prostate arising in the context of different genetic backgrounds, we used cDNA microarray analysis to measure transcript abundance levels for approximately 8,300 genes in the prostate glands of five frequently studied strains of *Mus musculus*; C57BL/6, 129X1/Sv, BALB/c, FVB/N and DBA/2. Four biological replicates consisting of tissues pooled from groups of three individuals were generated to facilitate statistical analyses and control for individual variability (Figure 1). We employed a common reference pool design to control for technical differences in array construction and hybridization. The transcript level of each gene was measured as the ratio of the intensity of hybridization signal for a strain-specific experiment relative to that for the reference pool.

**Figure 2:**
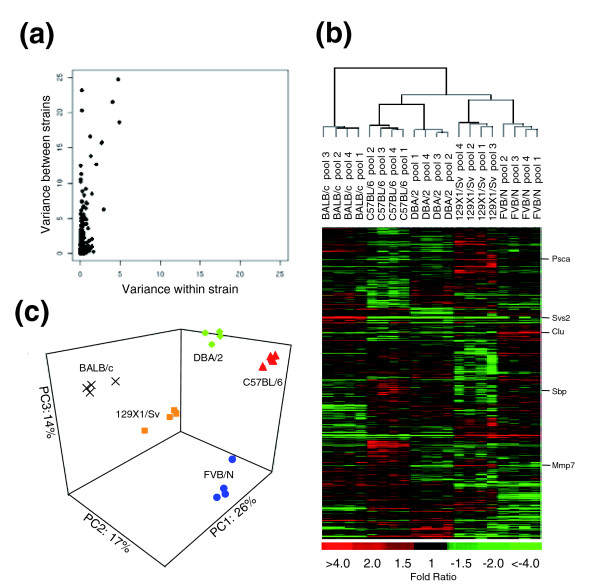
Prostate gene expression differences among strains. **(a) **Scatter plot of variance in gene expression levels between strains and within strains. **(b) **Average-linkage hierarchical clustering for the 932 differentially expressed genes among the five mouse strains (FDR <10%). Heat map colors reflect fold ratio values between sample and reference pool and mean-centered across samples. Columns represent biological replicates for each strain. Rows represent individual genes. Values shown in red are relatively larger than the overall mean; values shown in green are relatively smaller than the overall mean (see scale). Genes whose expression changes were confirmed by qRT-PCR, western blot or immunohistochemistry are listed. **(c) **Separation of the five strains in three-dimensional principal component space by applying PCA to the 932 genes with strain variance.

To determine the extent and magnitude of prostate gene expression variation between strains, we generated a one-way ANOVA table for each gene and compared the within-strain mean square (intra-strain replicates) to the between-strain mean square. As expected, the vast majority of genes exhibited low variance across the 20 array experiments. Furthermore, few differences were observed in the intra-strain comparisons, a result likely influenced by the pooling of samples to minimize the contribution of any individual mouse. However, comparisons of gene expression between strains identified substantial reproducible differences in the expression of many genes (range from 1.3 to 190-fold; Figure 2a). We used significance analysis of microarrays (SAM) procedures and applied a multiclass response *t*-test to identify genes whose expression in one strain significantly differed from the other four strains. Approximately 13% of the genes (932 genes) exhibited significant differential expression given a moderate estimate of false positive differences of 10%. The heat map revealed that the pattern of variability in transcript levels did not result from variations unique to a particular strain, but rather represents genetic variability across all five strains assessed (Figure 2b).

To explore the relationships between strains, we performed average linkage hierarchical clustering using all the genes (data not shown) and then using only the 932 genes that were differentially expressed between strains as determined by the SAM analysis (Figure 2b). The resulting dendrograms are identical, indicating that strain specific variation is not entirely explained by a small number of genes exhibiting large changes in gene expression. The expression patterns derived from prostates of the same strain are highly concordant and produce a consistent grouping of samples according to their strain of origin (Figure 2b). Overall, the samples are divided into three major branches: branch I is represented by BALB/c; branch II is represented by C57BL/6 and DBA/2; and branch III is represented by 129X1/Sv and FVB/N. Furthermore, within each branch, sub-branches clearly grouped pools according to strain.

In order to further characterize the relationship between strains, we performed principal components analysis (PCA) using the 932 differentially expressed genes (Figure 2c). The first four components explained 70% of the total variance. As expected, each of these informative components identified a subset of genes that discriminated between at least two of the strains. Taken together, these results show that strain-specific variation results from the differential expression of large numbers of genes and that this signal is stronger than the within-strain variability when using sample pools.

Among the expressed genes, those encoding pituitary tumor-transforming 1 (*Pttg1) *and adenylate cyclase-associated protein 1 (*Cap1) *were found to be differentially expressed between prostates of C57BL/6 and 129X1/Sv strains. Previous studies have found concordant strain-dependent differences in the expression of these genes in other mouse tissues [16]. Transcripts encoding several members of the histocompatibility complex also exhibited strain-dependent differences. Relative to other strains, H2-Ea is expressed highly in prostates of DBA/2 and BALB/c mice; H2-k is expressed highly in 129X1/Sv and C57BL/6; H2-Q1 is expressed highly in 129X1/Sv, FVB/N and C57BL/6; and transcripts encoding H2-D1 were least abundant in the C57BL/6 strain. Interestingly, the pattern of expression of this gene family did not correlate with the known H2 haplotypes of the strains, a finding also reported in a study evaluating strain-specific gene expression variation in the mouse hippocampus [17].

To identify differentially expressed genes unique to individual strains, we performed a pair-wise comparison of transcript abundance levels between each strain for a total of 10 pair-wise comparisons. The number of genes found to be differentially expressed between any two strains varied depending on the strains compared (Table 1). Strains 129X1/Sv and FVB/N exhibit the fewest differences in prostate gene expression (88 genes) whereas strains FVB/N and C57BL/6 exhibit the greatest number of transcript abundance differences (237 genes). Analyses of the promoter regions of these strain-variable genes did not identify sequence motifs that would suggest common regulatory mechanisms.

**Table 1:** Pairwise comparisons of mouse prostate gene expression between strains of *Mus musculus*

Strain	C57BL/6	129X1/Sv	FVB/N	BALB/c	DBA/2
129X1/Sv	124*	-	88	102	116
FVB/N	237	88	-	172	226
BALB/c	173	102	172	-	196
DBA/2	198	116	226	196	-

*Values represent the number of genes with significant differences in transcript abundance measurements between strains. Significance was defined as a SAM gene-specific q-value less than 0.05.

### Confirmation of strain-dependent differences in prostate gene expression

**Figure 3:**
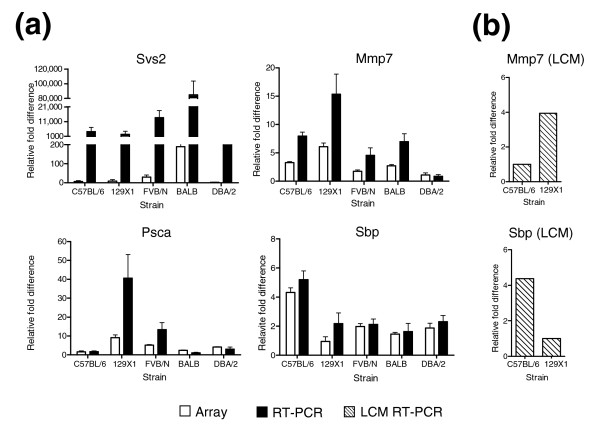
Analysis of strain-dependent differences in prostate gene expression by qRT-PCR. RNAs from preparations used in the **(a) **microarray analysis or **(b) **microdissected epithelium were reverse transcribed and amplified using qRT-PCR with primers specific for *seminal vesicle secretion 2 *(*Svs2*), *matrix metallopeptidase 7 *(*Mmp7*), *prostate stem cell antigen *(*Psca*) and *spermine binding protein *(*Sbp*). Ribosomal protein S16 expression levels were used to normalize qRT-PCR data. Normalized results are expressed relative to the lowest expressing value. Error bars indicate the standard deviation of four biological independent replicates. qRT-PCR for microdissected epithelium is represented by one sample per strain for each gene. White bars denote measurements from the microarray analysis. Black bars denote measurements generated by qRT-PCR from whole prostate. Diagonal lines denote measurements generated by qRT-PCR from microdissected prostate epithelium.

**Figure 4:**
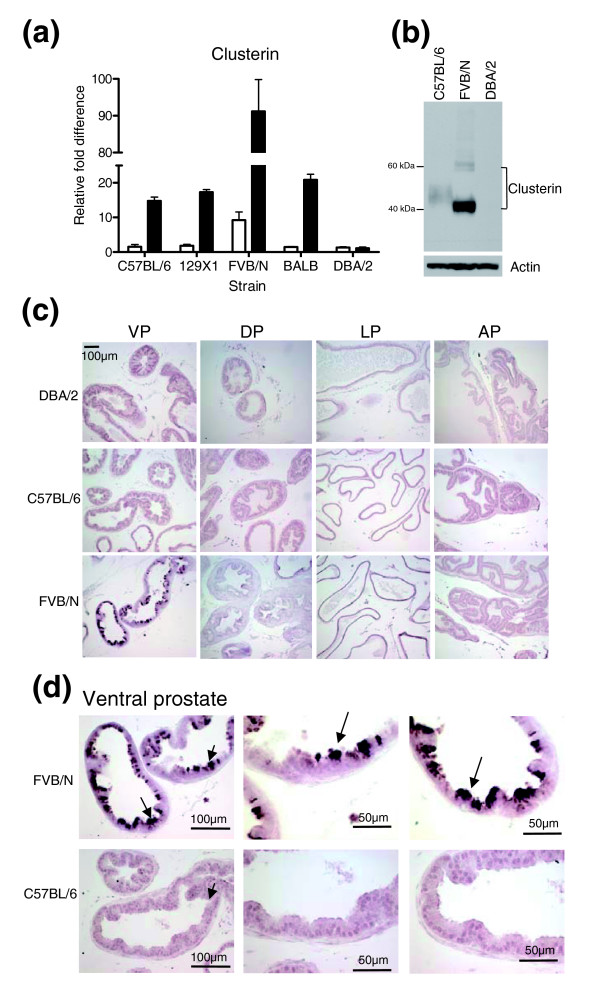
Clusterin is highly expressed in the FVB/N strain. **(a) **qRT-PCR measurement of *Clusterin *RNA in prostate preparations used in microarray analysis. White bars are the data from the microarray experiments and black bars are values generated by qRT-PCR. **(b) **Western blot analysis of clusterin in the ventral prostates of FVB/N, DBA/2 and C57BL/6 mouse strains. Ventral prostate tissue (pool of three ventral prostates per lane/strain) was prepared and equal amounts of protein were resolved by SDS-PAGE and probed with anti-clusterin antibody. Antibody against β-actin was used as a loading control. **(c, d) **Immunohistochemical analysis of paraffin sections from dorsal prostate (DP), lateral prostate (LP), anterior prostate (AP) (c) and ventral prostate (VP) lobes (c, d) of 8-9 week old mice from FVB/N, DBA/2 and C57BL/6 strains. Sections were stained with anti-clusterin antibody. Clusterin immunoreactivity is most intense in the apical region of the secretory epithelial cells from the ventral prostate (arrow).

Several genes exhibiting strain-dependent differences in prostate expression have been studied in the context of prostate development (for example, *Sbp*), androgen regulation (*Fabp5*, *Odc*), tumorigenesis (for example, *Psca*, *Azgp1*, *Apod*, *Mmp7*, *Egf*, *Mgst1*, *Clusterin*), and the progression of metastatic cancer (*Cxcl12*, *B2m*, H2 family members) [8]. To confirm the microarray results, we selected several of these genes for analysis by quantitative real-time reverse-transcription PCR (qRT-PCR). Primer pairs specific to *Svs2*, *Psca*, *Mmp7*, *Spb *and *Clusterin *were used to quantify transcripts in the same RNA samples used in the microarray experiments (Figure 3a; Figure 4a for clusterin). We measured transcripts encoding the housekeeping gene encoding ribosomal protein S16 to normalize the qRT-PCR data. From the microarray results, S16 expression did not vary significantly between strains.

Overall, the qRT-PCR transcript measurements for the five genes tested were in good agreement with the microarray data, though the magnitude of relative fold differences in the qRT-PCR assay was greater compared to the microarray results. This observation is partly due to intrinsic limitations in the microarray experimental design, where transcript levels were measured as the ratio between an experimental sample (strain sample) relative to that for the reference sample (pool of all strains). The expression of *Mmp7 *varied between 5- and 15-fold between strains with the greatest difference observed in a comparison of 129X1/Sv and DBA/2 mice (Figure 3a). The expression of *Psca *varied up to 40-fold between strains and the expression of *Clusterin *was at least 70-fold greater in the FVB/N mice relative to any other strain.

### Assessments of strain-associated variation in prostate cellular composition and cell type-specific gene expression

We hypothesized that strain-specific disparities in the ratios of cell types within the prostate gland could be reflected as measurable differences in transcript levels. The rodent prostate is composed principally of luminal secretory epithelium, basal epithelium, and a stroma consisting primarily of fibroblasts and smooth muscle, with a smaller component of endothelium, nerve cells, neuroendocrine cells, and inflammatory infiltrates. Since our transcript profiling studies were performed using whole prostates containing mixtures of the various cell types, we could not exclude the possibility that differences in gene expression between strains were a result of differences in cell type ratios between strains. To address this, we performed an *ad hoc *analysis using two prostates per strain, and calculated the percentage of prostate area occupied by stroma and epithelium for each lobe. Based on the estimated effect sizes and the corresponding *p *values, we did not identify significant strain-associated differences in the ratios of cell types between strains (data not shown).

To further confirm that prostate gene expression differences arise from intrinsic genetic variation and not cell ratio effects, we microdissected secretory epithelium from two strains: C57BL/6 and 129X1/Sv. We measured the transcript levels for two genes, *Sbp *and *Mmp7*, that exhibited strain-associated differences in the microarray studies. As shown in Figure 3b, transcript levels of *Mmp7 and Sbp *were four-fold higher and four-fold lower, respectively, in microdissected epithelium from 129X1/Sv relative to C57BL/6. These findings are in agreement with the differences in transcript levels observed for these genes in the analyses of whole prostates from these strains (compare Figures 3a and 3b). Together, these results support the conclusion that differences in prostate gene expression between strains, at least for the genes independently assessed in microdissected epithelium, represent an intrinsic cellular property rather than possible differences in prostatic cell type ratios between strains. Furthermore, the experimental design and microarray methods are capable of identifying transcript abundance differences between strains for genes expressed in a cell type- and lobe-specific manner (for example, *Sbp *[18,19]), even when diluted by mRNAs from all lobes and multiple cell types. However, it is likely that subtle, yet biologically relevant alterations in constituents of the stroma and glandular microenvironment also exist between strains. Identifying these differences will likely require detailed cell type-specific assays.

### Strain-associated differences in prostate protein expression

We next sought to determine if strain-associated differences in prostate transcript levels were reflected by concordant differences in protein expression. We chose to evaluate protein levels of clusterin, which is encoded by a gene studied extensively in the context of prostate carcinogenesis and therapy resistance [20-22]. Clusterin, also known as testosterone-repressed prostate message 2 (*TRPM-2*), is of particular interest in view of active efforts to target its expression as a treatment for human prostate cancer [20]. Although the function(s) of clusterin remains somewhat enigmatic, recent studies indicate that antiapoptotic effects are mediated in part through direct interactions with activated Bax [22]. We have previously shown that clusterin expression is increased in tumors developing in mice with a prostate specific deletion of the *Pten *tumor suppressor gene [23]. Microarray hybridization and qRT-PCR quantified clusterin transcripts at levels ten-fold or greater in prostates of FVB/N mice relative to all other strains (Figure 4a). A western blot analysis using ventral prostate protein extracts detected higher clusterin levels in prostates of the FVB/N strain when compared with DBA/2 and C57BL/6 strains (Figure 4b). We next performed immunohistochemistry to determine the cellular localization of clusterin expression. With the exception of the ventral lobe, we did not detect major differences in clusterin expression between mouse strains. However, substantially greater clusterin immunoreactivity was observed in the secretory epithelium of the ventral lobe of the FVB/N strain, relative to any other lobe and all other strains. Staining was particularly intense in the apical region of the epithelium, suggesting that the secretory form of clusterin is the predominant differentially expressed isoform in FVB/N ventral prostate epithelium (Figure 4c,d). Based on these results, we speculate that elevated clusterin levels may contribute to the enhanced rate of prostate tumor development and progression observed in the TRAMP FVB/N genotype.

### Biological pathway analysis of mouse prostate gene expression profiles

The substantial number of genes found to be differentially expressed in the prostates of different mouse strains suggested that specific groups of genes could share common regulatory mechanisms or participate in particular functional pathways. To address this possibility, we focused on differences between the C57BL/6 strain relative to other strains due to the reduced tumorigenicity observed in transgenic mouse prostate cancer models arising in the C57BL/6 background [14,15,24]. We used a method termed 'gene set test' (GST) in BioConductor that is analogous to the recently described gene set enrichment analysis (GSEA) algorithm [25] to determine if genes displaying relative differences in prostates of C57BL/6 mice were enriched in a database of biologically defined gene sets assembled by the Gene Ontology (GO) consortium. Only three of 258 gene sets, NADH dehydrogenase activity, NADH dehydrogenase (ubiquinone) activity, and phosphoinositide binding were statistically enriched in the C57BL/6 prostates (false discovery rate (FDR) ≤25%). While specific components of these pathways or networks could represent modifiers of the cancer phenotype, the results also suggest that influential genetic variation is broadly dispersed across functional biological pathways. This conclusion is tempered by acknowledged limitations to these studies that include the imperfect nature of algorithms used to determine gene enrichment and the fact that transcript measurements do not reflect the complete picture of biological pathways and networks.

### Gene expression variability in the human prostate: correlations with cancer phenotype

**Figure 5:**
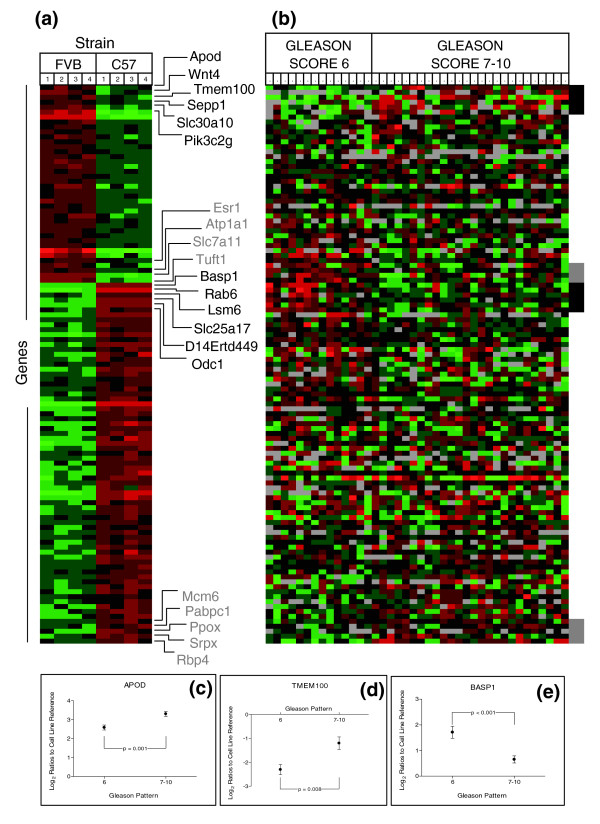
Mouse prostate strain-associated gene expression and analysis in human prostate tissues: FVB/N and C57BL/6. **(a) **Genes differentially expressed in prostates of FVB/N and C57BL/6 strains. Heat map colors reflect fold ratio values between sample and reference pool. Columns 1-4 represent biological replicates for each strain. Rows represent individual genes. Values shown in red are relatively larger than the overall mean; values shown in green are relatively smaller than the overall mean. **(b) **Transcript abundance levels in benign human prostate tissues associated with high grade (7-10) or low grade (≤6) adenocarcinomas for each gene determined to be altered in mouse strain comparisons where a corresponding ortholog was identified. Genes depected in (a) and (b) are in identical order. Black box (b) and text (a) represent genes with significant differential expression in the human datasets altered in the expected orientation. Gray box (b) and text (a) represent genes with significant differential expression in the human datasets altered in the opposite orientation. **(c-e) **Transcript alterations for selected genes in benign tissue samples associating with high (Gleason 7-10) and low (Gleason ≤6) prostate cancers. Plots represent the 95% confidence intervals of log_2 _expression ratios of tissues samples relative to a cell line reference.

**Figure 6:**
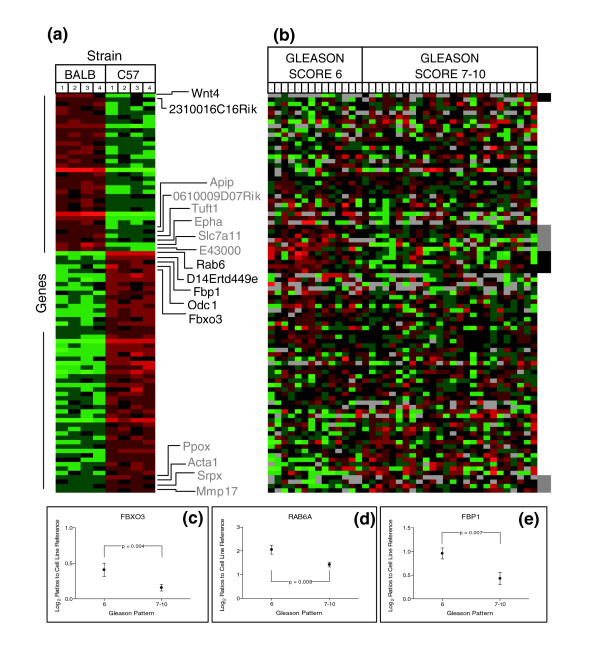
Mouse prostate strain-associated gene expression and analysis in human prostate tissues: BALB/c and C57BL/6. **(a) **Genes differentially expressed in prostates of BALB/c (BALB) and C57BL/6 (C57) strains. Heat map colors reflect fold ratio values between sample and reference pool. Columns 1-4 represent biological replicates for each strain. Rows represent individual genes. Values shown in red are relatively larger than the overall mean; values shown in green are relatively smaller than the overall mean. **(b) **Transcript abundance levels in benign human prostate tissues associated with high grade (7-10) or low grade (≤6) adenocarcinomas for each gene determined to be altered in mouse strain comparisons where a corresponding ortholog was identified. Genes depicted in (a) and (b) are in identical order. Black box (b) and text (a) represent genes with significant differential expression in the human datasets altered in the expected orientation. Gray box (b) and text (a) represent genes with significant differential expression in the human datasets altered in the opposite orientation. **(c-e) **Transcript alterations for selected genes in benign tissue samples associating with high (Gleason 7-10) and low (Gleason ≤6) prostate cancers. Plots represent the 95% confidence intervals of log_2 _expression ratios of tissues samples relative to a cell line reference.

Having established that consistent measurable differences in murine prostate gene expression occur in the context of genetic background, we next sought to determine if the orthologous genes were also variable in the human prostate, and whether the underlying normal gene expression levels, potentially representing quantitative traits, associate with aspects of human prostate carcinogenesis. We focused on transcript alterations between the C57BL/6 and FVB/N strains due to experimental evidence demonstrating that for the TRAMP model system of prostate cancer, the C57BL/6 genome delays cancer progression relative to an accelerated rate of carcinogenesis in other strains, including FVB/N [14]. We also focused on transcript differences between the C57BL/6 and BALB/c strains due to a recent report describing a reduced incidence of prostate adenocarcinomas in *Pten *deficient mice of a 129/C57 background relative to high rates of prostate carcinomas, up to 90% by 6 months, in *Pten *deficient mice of a 129/BALB/c background. These studies suggest the hypothesis that genes expressed highly in C57BL/6 prostates might function as inhibitors of carcinogenesis whereas genes expressed highly in other strains - relative to C57BL/6 - could function to promote or permit carcinogenesis. Direct comparisons of transcript abundance levels from prostates of the C57BL/6 strain against FVB/N and C57BL/6 against BALB/c identified 237 and 173 genes with significant differences, respectively (Table 1; Figures 5a and 6a).

We next measured the transcript abundance levels of these variable murine prostate genes in human prostate tissues. Based on the TRAMP mouse model data, we hypothesized that if genes expressed highly in C57BL/6 relative to FVB/N prostates (designated C57-High) retard aspects of carcinogenesis, they would be down-regulated in the prostates of those individuals shown to have aggressive prostate cancers, and if genes expressed highly in FVB/N relative to C57BL/6 prostates (designated C57-Low) promote aspects of carcinogenesis, they would be elevated in the prostates of individuals with aggressive prostate cancers. Similar reasoning was applied to genes differentially expressed between BALB/c and C57BL/6 prostates.

We analyzed data reported by Lapointe *et al*. [26] that generated independent gene expression profiles from matched pairs of benign and neoplastic human prostate tissues accompanied by pathological criteria of tumor aggressiveness according to the Gleason grading system. This human dataset contained orthologs for 113 of the 237 genes with differential expression in C57BL/6 relative to FVB/N prostates, and 91 of the 173 genes with differential expression in C57BL/6 relative to BALB/c prostates. We specifically focused on gene expression in the benign tissue of each human prostate sample as a potential measure of an underlying predisposition to cancer phenotypes reflected by cancer grade: low pathological grade (Gleason ≤6) versus cancers of higher grade (Gleason 7-10). In agreement with this hypothesis, seven genes expressed highly in C57BL/6 prostates were measured at significantly lower levels in prostates with high-grade cancers relative to prostates with low-grade cancers (for example, *BASP1*) (Figure 5b-e).

Six genes expressed relatively highly in FVB/N prostates (for example, *ApoD*) exhibited significantly higher transcript levels in the prostates containing high-grade cancers relative to prostates with low-grade cancer (Figure 5). Apolipoprotein D (*APOD*) is a member of the lipocalin superfamily of protein transporters that is implicated in the pathogenesis of neurodegenerative diseases and is regulated by androgens in both breast and prostate cells [27,28]. Studies of prostate cancer have demonstrated elevated APOD protein levels in prostate intraepithelial neoplasia and prostate carcinoma [29], but associations between *APOD *polymorphisms, or APOD expression in benign epithelium in the context of cancer phenotypes have not been reported. Two recent studies of the *Drosophila *ApoD ortholog, *GLaz*, provide context for the potential influence of ApoD expression on cytoprotection and cell survival [30,31]. Overexpression of Glaz increased resistance to stresses that included starvation, hyperoxia and hypoxia, and resulted in the extension of organismal lifespan [30]. Conversely, loss of *GLaz *resulted in the reduction of *Drosophila *stress resistance and lifespan, consistent with APOD being part of a defense system that is activated in the setting of oxidative stress, or incited by exogenous environmental factors or intrinsic events such as aging or neoplasia [31].

Counter to our hypothesis, several genes exhibited significant expression differences inversely associated with the human prostate cancer grade-status predicted by the mouse phenotypes. For example, transcript levels of four C57-Low genes (for example, *Esr1*) in benign prostate tissues were significantly associated with low-grade cancers and five C57-High genes (for example, *Rbp4*) were expressed at greater levels in benign tissue associated with high-grade relative to low-grade prostate cancers (Figure 5b). Similar results were observed for genes differentially expressed between BALB/c and C57BL/6 prostates when evaluated in benign tissues from prostates with high-grade cancers relative to prostates with low-grade cancers (Figure 6).

One possible explanation for these findings centers on different tumor initiating mechanisms (and pathways) that subsequently interact with different intrinsic intracellular and extracellular 'host' gene expression programs. For example, although studies of mouse prostate tumorigenesis demonstrate that the C57 strain appears to reduce tumorigenesis caused by *p53/Rb *(TRAMP model) or *Pten *alterations, the C57 background increases the development of prostate intraepithelial neoplasia lesions in mice with targeted deletions of the *Nkx3.1 *homeobox gene [32], and increases tumorigenesis in the setting of *ras+myc *expression, relative to other strains [33]. Thus, the complexities of the interactions between different tumor initiating events and host genomes are likely to be quite complex, a factor that could certainly influence the interpretation of human data where initiating events for any particular primary tumor are poorly defined. As heritable differences provide ample opportunities for mutations in growth control genes to exert differential effects depending upon the inherent wiring of the altered cell or the surrounding micro- and macroenvironment, improved sub-classifications of human prostate tumors that associate with specific oncogenenic events, such as the recently reported TMPRSS2-ERG fusions [34], may assist in defining consistent associations. In support of this concept are data demonstrating that a polymorphism in the promoter region of the *MDM2 *gene enhances MDM2 expression, attenuates p53 tumor suppressor function and accelerates tumor progression rates of both hereditary and sporadic human cancers arising in the context of p53 alterations [35].

## Conclusion

The quantification of tissue gene expression represents a measure of phenotypic variation at the molecular level that can be ascertained as comprehensive profiles reflecting active biological pathways. Exploiting gene expression information as quantitative traits (QTL) has facilitated large-scale efforts to identify genomic loci that contribute to complex phenotypes and diseases [36]. In this study we have found substantial reproducible gene expression differences in the murine prostate that associate with genetic background. Large strain-specific differences in tissue gene expression are not unique to the prostate. We have previously demonstrated that transcript abundance levels in the liver differ between mice of different strains [37]. Studies of strain-dependent differences in the development and progression of murine breast carcinoma have delineated gene expression differences in tumors arising in the context of genetic background [8], but it is likely that many of these differences were present in the breast tissues prior to cancer development. Indeed, mouse strains with high- or low-metastatic genotypes are reportedly distinguishable using gene expression measurements from benign breast tissue [38].

These data emphasize that, in addition to the initiating incident, context may be important for the process of carcinogenesis. Importantly, the predisposition to a detrimental sequence of events leading to pathology might be determined through assessments of the expression program operative in the normal state between different individuals. Thus, as with studies associating disease predisposition with variations in DNA polymorphisms, copy numbers, and epigenetic marks, studies of variation in gene expression in benign tissues, optimally with a tissue or cell type-specific focus, could provide insights into cancer predisposition and gene-environment interactions.

## Materials and methods

### Animal work and RNA preparation

Male mice 7 weeks of age were purchased from the Jackson Laboratory (C57BL/6J, 129X1/SvJ and FVB/NJ) or the Charles River Laboratories (BALB/cAnNCrl and DBA/2NCrl), maintained in a barrier facility and cared for in accordance with approved IACUC protocols. Following shipment, mice were acclimated to a common temperature, day-night cycle, and diet for 12 days to minimize environmental differences. Twelve mice from each of five strains, C57BL/6J, 129X1/SvJ, FVB/NJ, BALB/cAnNCrl and DBA/2NCrl (abbreviated C57BL/6, 129X1/Sv, BALB/c, FVB/N and DBA/2, respectively), were randomized for the day and time of sacrifice. Following halothane anesthesia, mice were sacrificed by cervical dislocation. Prostates were rapidly excised, and dorsal, lateral, ventral and anterior lobes were separately dissected and snap-frozen in liquid nitrogen and stored at -80°C. To control for individual variability [39], the dissected prostate lobes from mice of each strain were divided into four pools of three mice each and combined for total RNA isolation. Prostate tissues were homogenized using a Polytron and total RNA was isolated using the RNeasy extraction kit (Qiagen, Valencia, CA). Due to the limited concentration of total RNA obtained, specifically from the lateral lobe, equal amounts of total RNA (0.7 μg) from each of the four pooled lobes/strain were combined to produce secondary pools, and amplified with the MessageAmp™ aRNA kit (Ambion, Austin, TX). Amplified RNA from each secondary pool (four pools per strain) was hybridized separately to cDNA microarrays for a total of 4 independent biological replicates per strain (12 mice, 4 pools of 3 mice per strain for a total of 4 microarray experiments per strain; Figure 1). A common reference sample was created by combining equal amounts of RNA from all the samples from all strains.

### Probe construction, microarray hybridization and data acquisition

The amplified RNA was used as template for cDNA probe synthesis followed by hybridization to a custom mouse prostate cDNA array (mPEDB array) composed of approximately 8,300 genes expressed in the developing and adult mouse prostate [40]. The 20 microarrays used for the experiment were all from the same printing batch. cDNA probes were made from 2 μg of amplified RNA in a reaction volume of 30 μl containing 170 ng random hexamer primers, 0.2 mM 5-(3-aminoallyl)-2-deoxyuridine-5-triphosphate (amino acid-dUTP; Sigma-Aldrich, St. Louis, MO), 0.3 mM dTTP, 0.5 mM each dATP, dCTP, and dGTP, and 380 units of Superscript II reverse transcriptase (Invitrogen, Carlsbad, CA) incubated at 42°C for 120 minutes. After RNA hydrolysis, purified cDNA was combined with either Cy3 or Cy5 monoreactive fluors (Amersham Pharmacia, Piscataway, NJ) that covalently couple to the cDNA incorporated aminoallyl linker in the presence of 50 mM NaHCO3 (pH 9.0). The coupling reaction was quenched with hydroxylamine and reference and experimental probes were combined, filtered, and competitively hybridized to microarrays under a coverslip for 16 h at 63°C. Slides were washed sequentially with 1× saline sodium citrate (SSC)/0.03% sodium dodecyl sulfate (SDS), 1× SSC, 0.2× SSC, 0.05× SSC, and spun dry. To account for dye bias, half of the biological experimental samples per strain were labeled with Cy3 dye and the reference with Cy5 dye, and in the other half the labeling was inversed.

### Microarray data collection and analysis

Fluorescent array images were collected for both Cy3 and Cy5 using a GenePix 4000B fluorescent scanner (Axon Instruments, Foster City, CA, USA). The image intensity data were gridded and extracted using GenePix Pro 4.1 software. For each array spot, the expression levels of the two fluorophores were obtained by subtracting median background intensity from median foreground intensity. A gene was only considered expressed if the fluorescence intensity of the corresponding spot was at least six foreground pixels greater than four standard deviations above background in at least half of the arrays per strain. Array spots not meeting these criteria were designated NA. For each gene, the logarithm base 2 ratios (referred henceforth as log ratios) of the two channels were calculated to quantify the relative expression levels of genes between the experimental and reference samples.

To allow for inter-array comparisons, each array was normalized to remove systematic sources of variation. This was accomplished by means of a print-tip-specific intensity-based normalization method [41]. A scatter-plot smoother, which uses robust locally linear fits, was applied to capture the dependence of the log ratios on overall log-spot intensities. The log ratios were normalized by subtracting the fitted values based on the print-tip-specific scatter-plot smoother from the log ratios of experimental and control channels. After normalization, spots were removed from further analysis if they had more then 3 NA values or if they were in the lower third quantile of abundance across all 20 arrays.

To identify genes that varied among strains of mice, log_2 _ratio measurements were statistically analyzed using the SAM procedure [42]. A multiclass response *t*-test was used to determine whether the gene expression of one strain significantly differed from the other strains giving a moderate estimate of false positive genes of 10% (FDR). When differences in the expression of genes were examined using SAM software, 932 genes were statistically differentially expressed across the five strains of mice. Transcriptional profiles of the five mouse strains were also compared with average-linkage hierarchical clustering and Principle Components Analysis (PCA.) Average linkage hierarchical clustering was used to cluster the samples using all the genes or only those that were differentially expressed. To further characterize the relationships between the strains, we performed PCA on the 932 differentially expressed genes. All analyses were done using Bioconductor software [43].

### Comparisons of mouse prostate and human prostate gene expression for association with tumor phenotypes

To evaluate transcript abundance levels in benign human prostate epithelium that associated with cancer grade or stage, we focused on genes that were differentially expressed between prostates of C57BL/6 and FVB/N, and between C57BL/6 and BALB/c mouse strains. Based on studies of the TRAMP and *Pten *prostate cancer models, we designated the C57BL/6 strain as 'prostate cancer-inhibiting' and the FVB/N and BALB/c strains as 'prostate cancer-permitting'. The SAM algorithm was used to identify the 237 genes differentially expressed between C57BL/6-FVB/N and the 173 genes between C57BL/6-BALB/c strains using a FDR of <5%.

We next investigated whether differences in gene expression patterns of benign prostate tissues adjacent to prostate cancers were associated with Gleason pattern or patient outcomes. We evaluated a study by Lapointe *et al*. [26] that profiled benign tissues matched with prostate adenocarcinomas of defined Gleason grades. The data were derived from spotted cDNA microarrays and we normalized the data by fitting a print-tip specific Lowess curve to the log-intensity versus log-ratio plot. Normalized log_2 _ratio data were filtered to include clones with orthologs to the Mouse Prostate Expression Database (MPEDB) array. Out of the 237 and 173 genes that were identified as differentially expressed (q-value of <5%) between C57BL/6 and FVB/N, and between C57BL/6 and BALB/c, respectively, 113 and 91 had sufficient information to test in the Lapointe *et al*. data. An unpaired two-sample *t*-test was used to determine the association between expression level in the benign tissue and the corresponding Gleason pattern cancers adjacent to the normal samples. The R-code for this analysis can be found online at the MPEDB website in the supplementary data section [44].

### Biological pathway analysis

In order to identify specific biological pathways that exhibit strain specific variation, we utilized the GST method available in BioConductor, an algorithm similar to the GSEA method reported by Subramanian *et al*. [25]. Briefly, t-statistics comparing the expression levels of genes between the C57BL/6 and all other strains were used as input to the GST algorithm. All other settings were kept at their default status and the pathways were obtained from the GO database [45]. We defined a pathway as showing strain-specific variation if its corresponding FDR was less than 25%.

### Microdissection of luminal epithelium

Frozen sections (7 μm) were cut from snap-frozen mouse prostate glands and immediately fixed in cold 95% ethanol. After fixing, the slides were washed in deionized RNase-free water, stained with Mayer's hematoxylin for 30 seconds, followed by another water wash. The sections were then dehydrated with two one-minute washes in 100% followed by two five-minute changes in Xylene. Approximately 4,000 luminal epithelial cells were captured from the ventral, dorsal, lateral and anterior prostate lobes from 3 independent animals for a total of 12,000 cells per lobe using the Arcturus PixCell II instrument (Arcturus, Mountain View, CA, USA). Digital photos were taken of tissue sections before, during, and after LCM and assessed independently to confirm the cell type-specificity of the captured cells. Total RNA was extracted using the Picopure RNA isolation kit (Arcturus) according to the manufacturer's specifications. The extracted RNA was subjected to two rounds of amplification using the MessageAmpTM aRNA kit (Ambion).

### Quantitative RT-PCR

RNA from microdissected epithelium and from preparations used in the microarray analysis were used as template for qRT-PCR. We used 200 ng of amplified RNAs or 20 ug of total RNAs to generate cDNAs. SYBR GREEN real-time PCR was performed as previously described [39]. Primers to ribosomal protein S16 were used to normalize cDNA loading. The sequences of the primers used in this study were: *S16 *forward, 5'-AGGAGCGATTTGCTGGTGTGGA-3', *S16 *reverse, 5'-GCTACCAGGCCTTTGAGATGGA-3'; *Psca *forward, 5'-GCCTGGTAGAGGCTGAGATG-3', *Psca *reverse, 5'-ATCATCTCCTGGGACTCCTG-3'; *Clusterin *forward, 5'-TTTATGGACACAGTGGCGGA-3', *Clusterin *reverse, 5'-GCTTTTCCTGCGGTATTCCTG-3'; *Mmp7 *forward, 5'-CTGATGATGAGGACGCAGGA-3', *Mmp7 *reverse, 5'-ATTCATGGGTGGCAGCAAAC-3'; *Svs2 *forward, 5'-TCAGAAAGGCCGTCTCAGTT-3', *Svs2 *reverse, 5'-AGCTGCTTCGTCACTTCCTC-3'; *Sbp *forward, 5'-TGGAAACGATGATGATGACC-3', *Sbp *reverse, 5'-TGTGGAGATGCAGGACTGAG-3'.

### Western blot analysis

Prostates from three animals per strain were rapidly excised, and ventral lobes were separately dissected. Protein extracts were prepared by homogenizing and sonicating each ventral prostate lobe in 500 μl of T-PER buffer (Pierce Biotechnology, Rockford, IL, USA) and a cocktail of protease inhibitors (Roche, Indianapolis, IN, USA). Pooled ventral lobe lysates (50 μg) were separated by SDS-PAGE followed by western blot analysis using antibodies recognizing clusterin (M-18, Santa Cruz Biotechnology, Santa Cruz, CA, USA) and actin (I-19, Santa Cruz) at 1:200 and 1:400 dilutions, respectively.

### Immunohistochemistry

Formalin-fixed, paraffin-embedded mouse prostate tissue sections were deparaffinized, and endogenous peroxidase activity was blocked with 3% H_2_O_2 _for 5 minutes. Antigen was retrieved by steam heating with 10 mM citrate buffer (pH 6.0) for 20 minutes. For the detection of clusterin, a rabbit polyclonal anti-clusterin antibody (H330; Santa Cruz Biotechnology) was used at 1:1,000 dilution in blocking solution, and the sections were incubated for 1 h at room temperature. After washing, the slides were incubated for 30 minutes with biotinylated species-specific secondary antibody diluted 1:500 (Vector Laboratories, Burlingame, CA, USA), washed, then incubated with avidin-peroxidase complex (ABC, Vector Laboratories) for 30 minutes and visualized using VIP peroxidase substrate (Vector Laboratories). The sections were dehydrated, and permanently mounted. Species-specific IgG isotypes were added in lieu of primary antibody as controls, and these sections demonstrated no detectable staining. The expression of clusterin was assessed in four independent animals per strain.

### Tissue morphometry

To evaluate the percentage of each prostate lobe occupied by stroma, two formalin-fixed, paraffin-embedded prostates per strain were serially cut to generate 5 μm sections spaced 30 μm apart. Sections were stained with hematoxylin and eosin and the total tissue areas and stroma areas were separately calculated from each prostate lobe using ImageJ image analysis software [46]. Six measurements were taken from each of the four lobes for each of the five strains. To determine overall differences between strains, we fit the following model:

Y_ijk _= μ + Strain_i _+ Lobe_j _+ ε_k_

where Y_ijk _is the percentage of stroma area observed from mouse K, lobe j, strain i.

### Data

Microarray data for this study have been deposited in the Gene Expression Omnibus [47] under accession GSE5962.
